# Higher serum levels of short-chain fatty acids are associated with non-progression to arthritis in individuals at increased risk of RA

**DOI:** 10.1136/annrheumdis-2021-221386

**Published:** 2021-11-24

**Authors:** Klara Martinsson, Kerstin Dürholz, Georg Schett, Mario M Zaiss, Alf Kastbom

**Affiliations:** 1 Department of Biomedical and Clinical Sciences, Linköping University, Linköping, Sweden; 2 Department of Internal Medicine 3, Rheumatology and Immunology, Friedrich-Alexander-University Erlangen-Nürnberg (FAU) and Universitätsklinikum Erlangen, Erlangen, Germany; 3 Deutsches Zentrum Immuntherapie (DZI), Friedrich-Alexander-University Erlangen-Nürnberg (FAU) and Universitätsklinikum Erlangen, Erlangen, Germany

**Keywords:** arthritis, therapeutics, inflammation, arthritis, rheumatoid

Transition from the autoimmune to the clinical phase of rheumatoid arthritis (RA) is a critical step that is yet insufficiently understood. Identification of factors that facilitate the progression from this prodromal RA at-risk state to clinical RA may open new possibilities for preventive interventions. In this context, nutritional factors may be critical. Short-chain fatty acids (SCFAs) are intestinal microbial metabolites that result from nutritional fibre digestion and exert immune regulatory properties.^1^ SCFAs have shown to effectively inhibit the onset of experimental arthritis.^2^ Furthermore, serum butyrate levels decrease shortly before the onset of arthritis.^2^ Whether SCFA levels may play a role in the transition from the autoimmune to the clinical phase of RA in humans, however, has not been studied to date.

**Figure 1:**
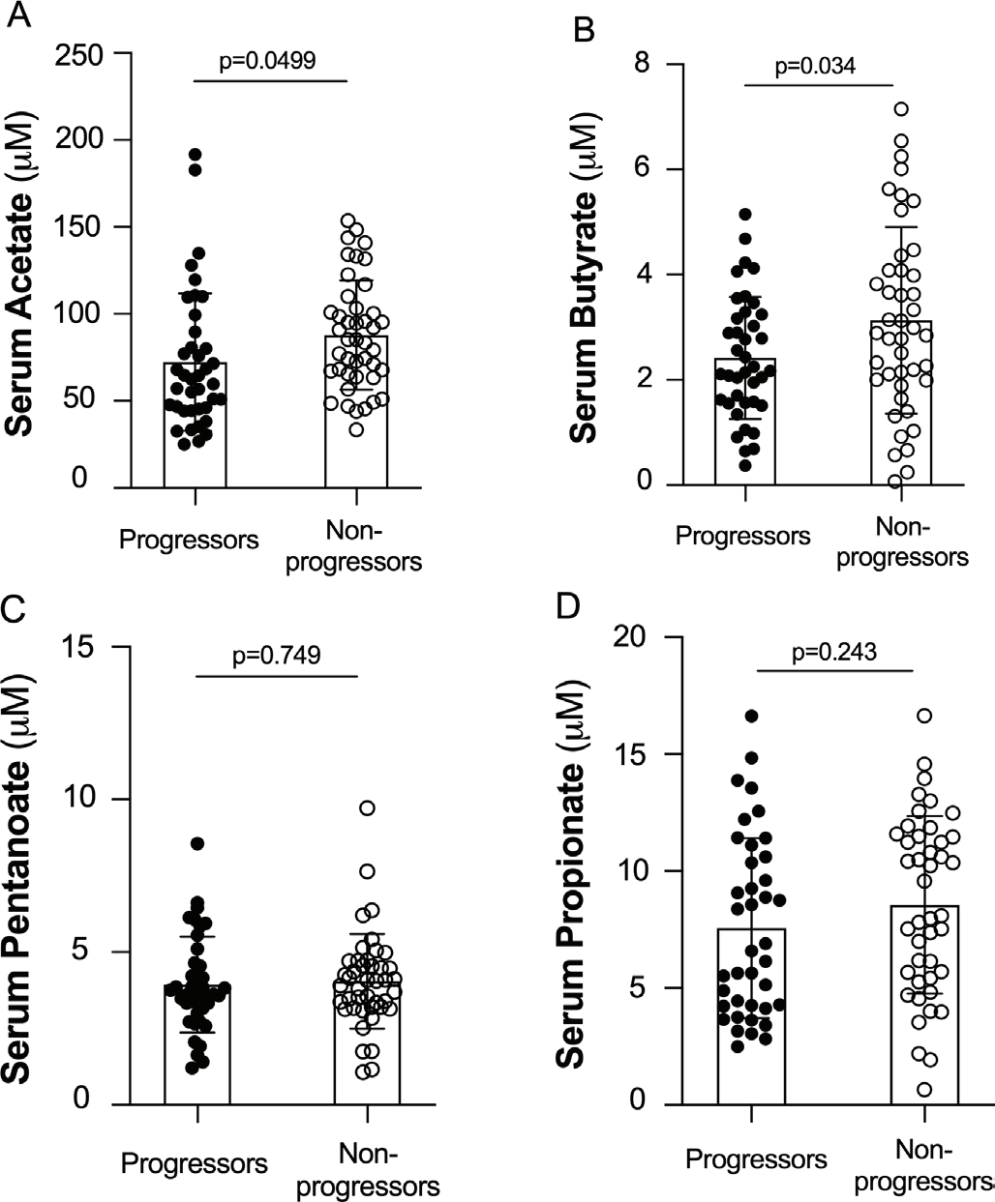
Baseline serum samples from rheumatoid arthritis at-risk individuals (ACPA+; musculoskeletal pain+) progressing (n=39) or not progressing (n=43) to arthritis in a prospective observational cohort study^3^ were analysed for (A) acetate, (B) butyrate, (C) pentanoate and (D) propionate levels. Bars represent means and error bars represent SD. ACPA, anti-citrullinated protein antibodies.

To address this concept, we measured serum SCFA levels in a prospective cohort of 82 individuals with an increased risk to develop RA.^3^ At inclusion, these individuals were positive for anti-citrullinated protein antibodies (ACPA) and had musculoskeletal pain but no clinical signs of arthritis (joint swelling). Baseline characteristics are shown in online supplemental table 1. Following a median follow-up of 72 months, 39 patients (48%) had developed clinical arthritis after a median of 6 months. Baseline serum samples were analysed for SCFA concentrations as previously described.^4^ At-risk individuals not progressing to arthritis had significantly higher mean baseline serum levels of total SCFA (ie, the sum of acetate, butyrate, propionate or pentanoate), butyrate and acetate as compared by t-test to individuals who progressed to arthritis (figure 1). In contrast, levels of propionate and pentanoate did not significantly differ (figure 1). Univariable Cox regression analyses revealed significant association between lower total SCFA levels and progression to arthritis (p=0.029), while for the individual SCFA, we found significant associations concerning butyrate (p=0.038) and acetate (p=0.039) levels, but not regarding pentanoate or propionate (online supplemental table 2). Statistical significance remained after adjusting for age, sex, symptom duration, rheumatoid factor status, ACPA levels and CRP levels (total SCFA p=0.030; butyrate p=0.009 and acetate p=0.045, online supplemental table 2).

10.1136/annrheumdis-2021-221386.supp1 Supplementary data

Butyrate levels inversely correlated with serum IgA-ACPA levels (r=−0.23, p=0.039), but not with IgG-ACPA or IgM-ACPA. No other SCFAs were significantly correlated with any ACPA subtype.

These data suggest that SCFA, in particular butyrate and acetate, influences the risk for the transition from the autoimmune to the clinical phase of RA. Although most p values would not remain significant after correction for multiple testing, the data are in line with previous findings in animal models^2^ and thus confirm our prespecified hypothesis. As SCFAs are produced by intestinal microbiota on fermentation of dietary fibres, our findings strengthen the concept that nutritional factors could influence the onset of RA. SCFAs are critical for the barrier function of the intestinal epithelium and thereby influences the migration of cells from the gut to the joints.^2^ Increasing SCFA levels by direct supplementation, fiber-rich diet or faecal transplantation to restore early dysbiosis thus represent potential strategies to inhibit the onset of arthritis.^4–6^ In this context, high-fibre diet has already shown to increase SCFA levels and decrease inflammatory burden in patients with established RA^4^ but has not been applied in a preventive setting. These data suggest that a state of high SCFA concentrations, which can be reached by dietary interventions such as high-fibre diet, may go along with a lower risk to progress to clinical arthritis in individuals with a high risk to develop RA.
