# Supplementary material for: Higher serum levels of short-chain fatty acids are associated with non-progression to arthritis in individuals at increased risk of RA
Source: Ann Rheum Dis. 2021 Nov 24;81(3):445–7. doi: 10.1136/annrheumdis-2021-221386 (PMC8862054; doi:10.1136/annrheumdis-2021-221386)
Supplement: Supplementary data [file annrheumdis-2021-221386supp001.pdf]

Supplementary Tables

Supplementary Table 1. Baseline characteristics of the TIRx cohort.

	**Patients, n=82**
Age, mean (SD), years	52 (14)
Female, n (%)	66 (81%)
Symptom duration	
0-6 months, n (%)	15 (18%)
6-18 months, n (%)	37 (45%)
>18months, n (%)	30 (37%)
RF positive, n (%)	24 (29%)
ACPA level	
Low (<3 *cutoff)	32 (39%)
High (≥3*cutoff)	50 (61%)
CRP, mg/L, mean (SD)	6 (6)
ESR, mm/h, mean (SD)	12 (10)
Shared epitope carrier n (%)	52 (63%)
Smoking	
Current smoker, n (%)	13 (16%)
Former smoker, n (%)	26 (32%)

RF= Rheumatoid Factor, ACPA= Anti-Cyclic Citrullinated Peptide antibodies. CRP= C reactive protein, ESR= Erythrocyte Sedimentation Rate.

Supplementary Table 2. Cox regression analyses for baseline short chain fatty acid levels versus progression to arthritis.

	**Univariable analysis**		**Adjusted analysis[a]**	
	HR (95% CI)	p-value	HR (95% CI)	p-value
Total SCFA (μM)	0.988 (0.978 - 0.999)	0.029	0.988 (0.978 - 0.999)	0.030
Acetate (μM)	0.989 (0.987 - 0.999)	0.039	0.989 (0.978 - 1.000)	0.045
Butyrate (μM)	0.791 (0.634 - 0.987)	0.038	0.705 (0.543 - 0.917)	0.009
Pentanoate (μM)	0.924 (0.745 - 1.145)	0.469	-	-
Propanoate (μM)	0.973 (0.895 - 1.059)	0.529	-	-

[a] Adjusted for age, sex, symptom duration, rheumatoid factor status, ACPA levels, and CRP levels at sampling. CI: confidence interval, HR: hazard ratio, SCFA: short chain fatty acids
